# A Convenient and High-Efficient Laser Micro-Engraving Treatment for Controllable Preparation of Microstructure on Al Alloy

**DOI:** 10.3390/ma11112297

**Published:** 2018-11-16

**Authors:** Mingkai Tang, Yusheng Shi, Wenzhi Zhu, Nan Zhang, Lichao Zhang

**Affiliations:** School of Materials Science and Engineering, Huazhong University of Science and Technology, 1037 Luoshi Road, Wuhan 430074, China; wate8788@yahoo.com (M.T.); shiyusheng@hust.edu.cn (Y.S.); start919@126.com (W.Z.); pluto1347@126.com (N.Z.)

**Keywords:** Al alloy, laser micro-engraving, controllable preparation, surface microstructure

## Abstract

Surface microstructure preparation offers a promising approach for overcoming the shortcomings of Al alloy, such as poor friction resistance, low hardness and weak corrosion resistance to corrosive liquid. Though many methods for the surface microstructure preparation of Al alloy have been developed, it is difficult for most of the reported methods to regulate the as-prepared microstructure, meaning that the properties of Al alloy cannot be improved efficiently by the microstructure. Thus, the application of microstructure surface of Al alloy and microstructure preparation technology is severely limited. Aimed at this issue, a simple, convenient, high-efficient, low-cost micro-scale roughness structure construction approach that is suitable for engineering application (laser micro-engraving) was developed. The as-prepared microstructure on Al alloy surface formed by laser micro-engraving was investigated systemically. The morphology and formation mechanism of the microstructure were examined. Meanwhile, the effect of laser parameters on morphology, geometrical dimensions and composition of microstructure was investigated. The results indicate that the morphology of microstructure is affected by the overlap degree of molten pool greatly. When each molten pool does not overlap with others, successive individual pits can be constructed. When each molten pool overlaps with others for one time, successive overlapping pits will form. As the overlap degree of the molten pool further increases (overlapping with others for more than one time), the successive pits can become grooved. Because of the influence of laser beam pulse frequency and scanning speed on the diameter and distance of the molten pools, the morphology and geometrical dimensions of microstructure can vary greatly with laser parameters. As the laser beam scanning speed increases, the geometrical dimensions of as-prepared microstructure reduce significantly. In contrast, with the increase of laser beam pulse frequency, the geometrical dimensions change in a complicated manner. However, the chemical composition of microstructure is slightly affected by laser parameters. More importantly, a relationship model was successfully established, which could be used to predict and regulate the geometrical dimensions of microstructure treated by laser micro-engraving. Controllable preparation of microstructure on Al alloy is realized, leading that specific microstructure can be prepared rapidly and accurately instead of suffering from long-time experimental investigation in the future.

## 1. Introduction

Due to the excellent properties, such as high strength to weight ratio, high conductivity of electricity, good formability, non-ferromagnetic property and high corrosion resistance, aluminum alloy has an extensive application in many fields, including automobile, ship, aerospace, aviation, sensor and architecture, etc. [1,2,3,4]. Nowadays, Al alloys have been the most-widely-used alloy [5]. However, Al alloys also have some disadvantages, such as poor friction resistance, low hardness, bad corrosion resistance to corrosive solutions, resulting in the limitation of further application [6,7,8].

Because of the distinctive surface microstructure, plants and animals exhibit various peculiar characteristics [9,10,11]. For example, due to the micro/nanoscale hierarchical structures and wax on the surface of a lotus leaf, it is difficult for water and dust to stay on the surface [12]. Micron-sized grooved scales growing on shark skin are interlocked to form a natural non-smooth surface. The grooved scales can reduce vortices formation and lift the vortices off the surface [13]. Inspired by this, surface modification emerges. Through constructing a specific microstructure on a material surface, not only can the properties of the material be improved, but also unique function (superhydrophobicity, self-cleaning, anti-icing, light trapping, etc.) can be given to the material [14,15,16]. Undoubtedly, the shortcomings of Al alloy can be overcome by preparing specific microstructure on the surface, which attractes much attention [17,18]. Great effort has been focused on constructing rough structure on Al alloy surface. Nowadays, many methods have been developed to prepare microstructure on Al alloy, such as chemical etching, sol-gel, anodization, hot water immersion, electrodeposition and laser etc. [19,20,21]. For example, via hydrochloric acid etching and potassium permanganate passivation, Li et al. constructed micro/nano-scale terrace-like hierarchical structures on 6061 Al alloy surfaces [22]. Through anodization, a hierarchical micro-nanostructure was successfully fabricated on Al substrate by Zheng et al. [23].

Though various methods have been developed, they still face some limitations including rigorous process, time consuming and small-scale preparation [24,25]. Moreover, the as-prepared microstructure exhibits low mechanical strength, poor abrasion resistance and low adhesion force with substrate, which limits the application in many fields, and especially, in engineering. More importantly, surface microstructure on Al alloy prepared by most of the reported methods is random and irregular [17,26,27]. The evolution of morphology of the formed microstructure with process parameters is not comprehensively researched as well [28,29]. Therefore, it is difficult to rapidly and accurately prepare the expected microstructure that is able to improve the Al alloy properties. Controllably regulating the morphology and dimension of the as-prepared microstructure cannot be realized as well, which means that the microstructure can play an important role in improving the properties of Al alloy. This limitation confines the applications of microstructure surface of Al alloy and microstructure preparation technology.

In recent years, because of fast adaptability, high precision and cleanness of environment, laser technology has been widely applied in various fields [30]. It is used for surface treatment as well. For example, Hu et al. prepared micro pit on the surface of Al-Si alloy via laser treatment [31]. In this paper, aiming to realize accurate preparing expected microstructure and controllable regulating the morphology and dimension of the as-prepared microstructure, a simple, convenient, high-efficient, low-cost and suitable for engineering application micro-scale roughness structure construction approach combined with laser technology (laser micro-engraving) was developed. The microstructure on Al alloy prepared by laser micro-engraving surface was investigated systemically. The morphology and formation mechanism of the microstructure under different process conditions were studied. The effect of laser parameters on morphology, geometrical dimensions and composition of microstructure was investigated. The effect rule and mechanism were exposed. Moreover, the relationship between the geometrical dimensions of microstructure and laser parameters was clarified. A relationship model was successfully established, which could be used to predict and regulate the geometrical dimensions of microstructure prepared by laser micro-engraving.

## 2. Materials and Methods

### 2.1. Materials

The commercially available AA7075 Al alloy plates (thickness of 6 mm) were purchased from Alcan Alloy Products Co., Ltd. (Montreal, QC, Canada) (the chemical composition in mass percent: 0.4 Si, 1.2 Cu, 0.3 Mn, 2.1 Mg, 0.20 Cr, 5.1 Zn and the balanced Al). The plate was cut by wire-cut electrical discharge machining into the square pin of 20 mm × 20 mm. Then, the cut samples were grounded to polish mechanically with successive grades of emery papers from 500 grit to 1200 grit, to conduct the following treatment.

### 2.2. Laser Micro-Engraving

Laser micro-engraving was carried out by a commercially available portable laser engraving device (Chuanlei Laser Equipment Ltd., Shanghai, China) (YAG laser, laser beam wavelength of 1064 nm, laser beam pulse width of 20 ns, laser beam diameter of 80 μm, output power of 20 W). The surface of the samples was treated under different processing parameters. After treatment, all the samples were cleaned twice in ultrasonic bath by absolute ethyl alcohol (Baobo Ultrasonic Ltd., Hangzhou, China) at room temperature and each for 10 min.

### 2.3. Characterization

The morphology of the treated surfaces was observed by a Quanta 200 FEG environmental scanning electronic microscopy (ESEM) (FEI Ltd., Hillsboro, OR, USA). The dimension of the microstructure of the treated surface was measured on a Contour Elite I 3D optical surface profiler (Bruker Daltonics Inc., Billerica, MA, USA) purchased from Bruker. Measurement was conducted five times and the mean value was adopted. X-ray photoelectron spectroscopy (XPS) was carried out on a VG ESCALAB MKII spectrometer (Kratos Analytical Ltd., Manchester, UK) using an Mg Kα X-ray source (1253.6 eV, 120 W) as the X-ray excitation source.

## 3. Results and Discussion

### 3.1. Preparation Mechanism

**Figure 1:**
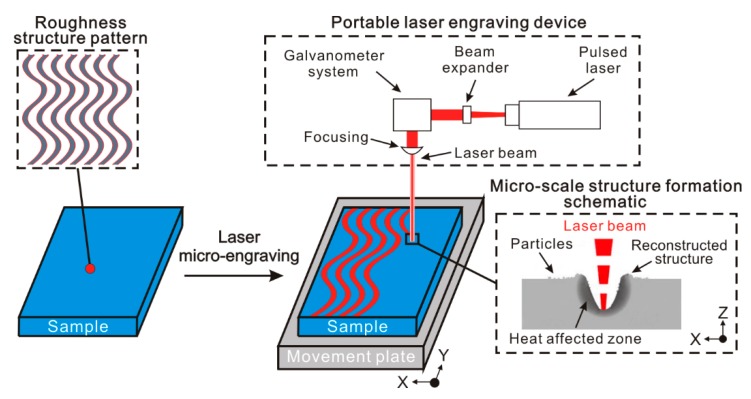
The treatment process and schematic of the micro-scale roughness structure construction approach (laser micro-engraving).

The treatment process and schematic of the micro-scale roughness structure construction approach are shown in Figure 1. Before treatment, according to the macroscopic morphology of the prepared roughness structure, the roughness structure pattern was designed. Due to the open-end design of the pattern, a roughness structure with complicated morphology can easily be prepared on the surface by this method. During laser micro-engraving treatment, laser beam will travel along with the pattern at a constant speed. From the micro-scale structure formation schematic, it can be easily found that the material of surface layer is melted rapidly when the laser beam continuously irradiates the surface, leading to the formation of a molten pool. Then, some melted material in the molten pool is vaporized and removed, which results in the formation of a concave structure and heat-affected zone. Simultaneously, because of the laser beam impact force, a part of melted material is squeezed out from concave structure and piles up together, which forms the reconstructed structure (convex structure). Furthermore, due to the different pressure between vaporized material and atmospheric, blasting and splashing phenomenon occurs in the molten pool. The splashing melted material will form particles in the air. These particles are randomly distributed on the reconstructed structure and untreated surface. Since the diameter of the laser beam used for portable laser engraving device is about 80 μm, the as-prepared structure is micro-scale in structure. Furthermore, the portable laser engraving device which is generally used to engrave the label of items is cheap and common. The efficiency of treatment is very high as well, for the scanning speed can be as high as 1 m/s. Therefore, the micro-scale roughness structure construction approach is convenient, low-cost and high-efficient. More importantly, because of no requirement for the thickness and area of the treated material, it is suitable for engineering application.

**Figure 2:**
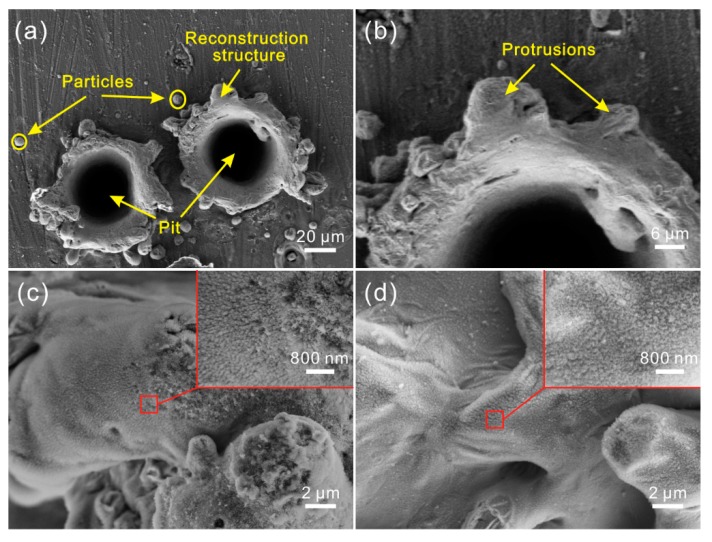
(**a**) SEM image of the microstructure on Al alloy surface treated by laser micro-engraving (laser beam pulse frequency of 10,000 Hz, laser beam scanning speed of 0 m/s); (**b**) high-magnification SEM image of the microstructure; (**c**) high-magnification SEM images of convex structure of the microstructure; (**d**) high-magnification SEM images of the pit.

The SEM image of the Al alloy surface after laser micro-engraving (laser beam pulse frequency of 10,000 Hz, laser beam scanning speed of 0 m/s) is shown in Figure 2a. Clearly, due to no movement of the laser beam, a circular pit was constructed on the Al alloy. Around the pit, irregular reconstruction convex structures could be easily observed. Moreover, some spherical particles were randomly distributed on the convex structure surface. From the high-magnification SEM image shown in Figure 2b, it was seen that some irregular protrusions were formed on the convex structure surface. Additionally, fold could be observed easily on the circular pit, which indicated that the formation of the pit was not mainly due to the material vaporization, but rather, the melted material squeeze induced by laser beam impact force. As shown in Figure 2c,d, it was obvious that the surfaces of the convex structure and pit were covered with a myriad of regular flocculent nanostructures, indicating that laser micro-engraving treatment not only constructs microstructure on Al alloy surface, but also forms nanostructure.

### 3.2. Morphology Evolution

According to the treatment process, the laser beam travels along a track which is set up at a constant speed during the treatment. Because of the pulse laser used for laser micro-engraving, as the laser beam moves, successive hemispherical molten pools with a constant distance form on the surface by each laser beam irradiation, which finally results in the formation of strip microstructure. As the distance of each molten pool changes, the morphology of the microstructure may greatly be affected. In the following, the strip microstructures with different molten pool spacing prepared by laser micro-engraving were investigated.

**Figure 3:**
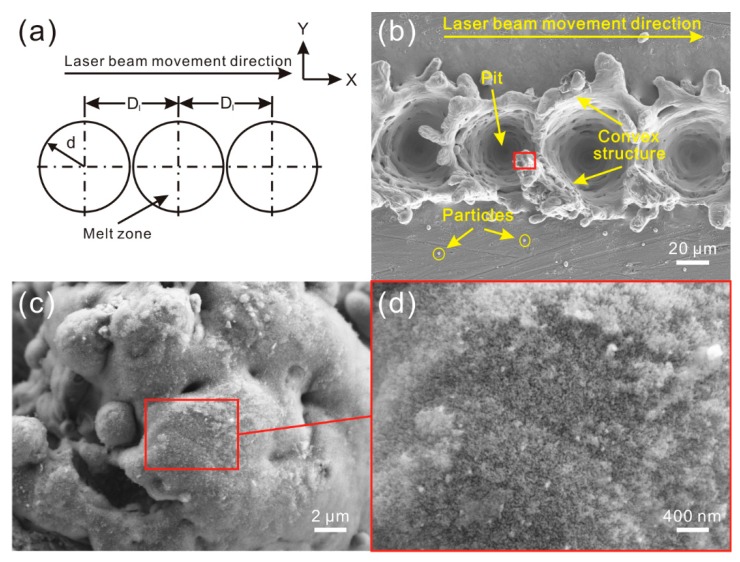
(**a**) Schematic of overlap degree of molten pools (no overlap of melting zone, i.e., D_1_ ≥ 2d); (**b**) SEM image of the microstructure on Al alloy surface (laser beam pulse frequency of 10,000 Hz, laser beam scanning speed of 0.4 m/s); (**c**,**d**) high-magnification SEM image of the microstructure.

When each molten pool did not overlap with others (D_1_ ≥ 2d) (Figure 3a), as shown in Figure 3b, successive and circular pits were constructed along the direction of laser beam movement on Al alloy surface. Each pit was individual and maintained the same diameter. Similar to the microstructure shown in Figure 2a, the pits were surrounded by the irregular convex structure. Moreover, some particles with different sizes could be observed. From the high-magnification SEM images shown in Figure 3c,d, flocculent nanostructure was formed on the microstructure surface.

**Figure 4:**
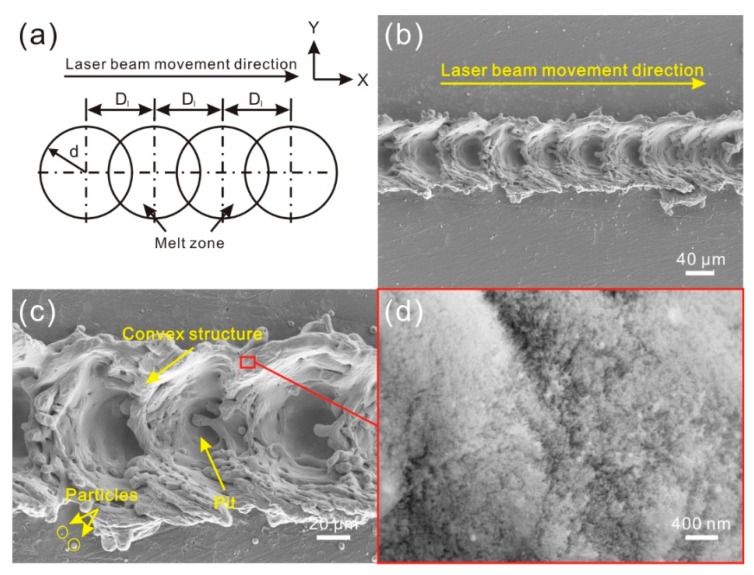
(**a**) Schematic of overlap degree of molten pools (overlap of each melting zone, i.e., d ≤ D_1_ < 2d); (**b**) SEM image of the microstructure on alloy surface (laser beam pulse frequency of 8000 Hz, laser beam scanning speed of 0.4 m/s); (**c**,**d**) high-magnification SEM image of the microstructure.

With the decrease of distance of the molten pools, the molten pools come into contact with each other. When each molten pool overlaps with others one time (d ≤ D_1_ < 2d) (Figure 4a), the morphology of the treated surface is as shown in Figure 4b. Though successive circular pits were prepared on the Al alloy surface, each pit contacted with the neighboring pit. As shown in Figure 4c, the pits were still surrounded by convex structure. However, along the direction of laser beam movement, the convex structure where was in the overlap zone was much lower than the structure on both side of the pits. It indicates that the overlap of molten pool leads the melted material of previous reconstructed structure in the overlap zone to be squeezed to the two flanks of the pits by the laser beam impact force induced by follow-up laser beam. From the high-magnification SEM image shown in Figure 4d, a similar phenomenon could be observed. Flocculent nanostructure was covered densely on the microstructure surface.

**Figure 5:**
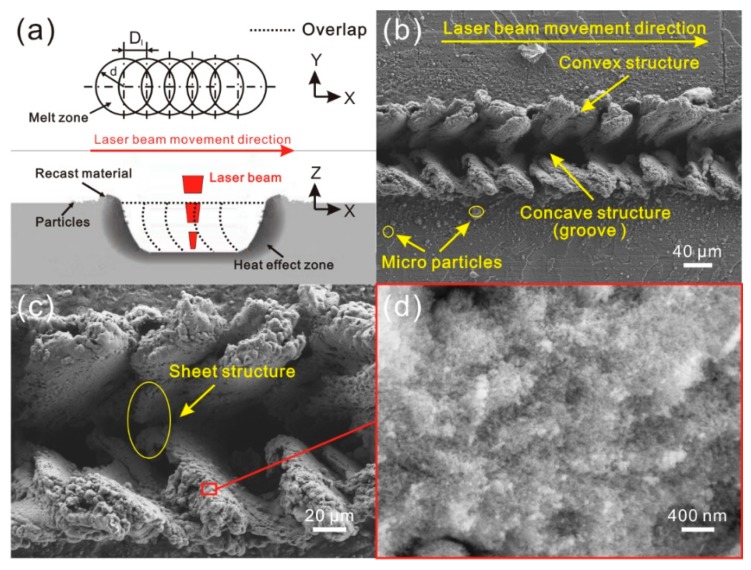
(**a**) Schematic of overlap degree of molten pools (multiple overlap of each melting zone, i.e., 0 < D_1_ < d/2); (**b**) SEM image of the microstructure on Al alloy surface (laser beam pulse frequency of 6000 Hz, laser beam scanning speed of 0.15 m/s); (**c**,**d**) high-magnification SEM image of the Al alloy surface.

As the distance of molten pools further reduced, the overlap degree of molten pools increased. As shown in Figure 5a, when each molten pool overlapped with others for more than one time (0 < D_1_ < d), the microstructure on Al alloy surface showed great change (Figure 5b). Strip groove was formed instead of successive circular pits on the surface. Moreover, the reconstructed sheet convex structures were arranged parallel to each other along the direction of laser beam movement. The angle between the sheet convex structure and strip groove was about 30°. Additionally, the number of the particles covered on the convex structures increased dramatically. As shown in Figure 5c, the sheet microstructure constructed by the remainder of the melted material that was not squeezed out appeared at the bottom of the strip groove, further indicating that the formation of the depression structure is mainly due to the melted material squeeze induced by laser beam impact force. As the distance of molten pool reduces, the times of laser beam impact (per unit area) increase. The effect of the laser beam impact on melted material is similar to plowing, leading to the aforementioned change of as-prepared microstructure. Meanwhile, the degree of blasting and splashing of the melted material also improves. From the high-magnification SEM image shown in Figure 5d, the strip groove surface was covered with flocculent nanostructure, indicating that the nanostructure can be formed on the Al alloy surface under different laser parameters (laser beam pulse frequency and laser beam scanning speed).

**Figure 6:**
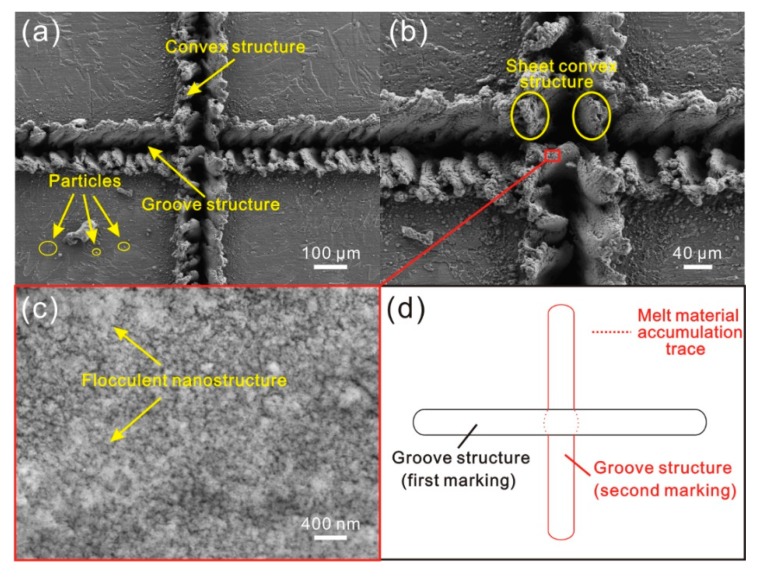
(**a**) SEM image of cross microstructure on Al alloy surface (laser beam pulse frequency of 6000 Hz, laser beam scanning speed of 0.15 m/s); (**b**,**c**) high-magnification SEM image of the microstructure; (**d**) schematic of the preparation process of cross structure.

In the following, the cross microstructure composed of strip microstructures was also investigated. The morphology of the cross microstructure is shown in Figure 6. Because of the same parameters, the strip grooves and convex structure were the same as the microstructures shown in Figure 6b. However, at the intersection area of the two strip grooves, the irregular sheet microstructure that was perpendicular to the horizontal strip groove was constructed. Moreover, compared with the other area, the untreated surface near the intersection area was covered with a much larger number of particles. As shown in Figure 6c, there were numerous flocculent nanostructures on the sheet microstructure surface. From the schematic of the preparation of cross microstructure shown in Figure 6d, it could be found that the melted material accumulated at the intersection area was affected by laser beam impact force again during the second laser micro-engraving treatment (constructing vertical strip groove). The melted material was squeezed into the horizontal strip groove, resulting in the formation of sheet convex microstructure. At the same time, due to the effect of the laser beam on the material at of the intersection area, the degree of blasting and splashing of the melted material increased.

**Figure 7:**
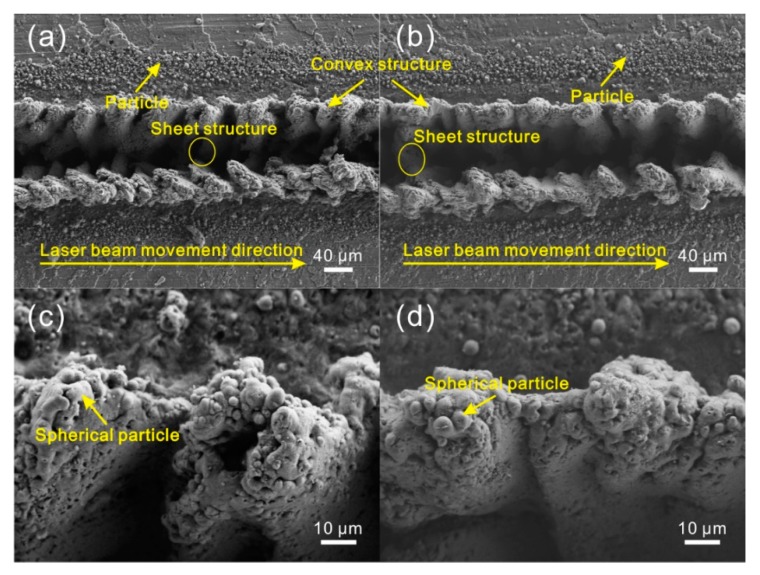
SEM image of the microstructure on Al alloy surface (laser beam pulse frequency of 6000 Hz, laser beam scanning speed of 0.15 m/s): (**a**) 2 times; (**b**) 4 times; (**c**,**d**) corresponding high-magnification SEM images.

The following part investigated the influence of laser micro-engraving times on the morphology of treated strip microstructure. When the laser micro-engraving times increased to 2 (Figure 7a,c), compared with the microstructure shown in Figure 5b,c (laser micro-engraving of 1 time), the morphology almost had no obvious change. The width of the strip grooves still remained about 60 μm. However, the size of the sheet microstructure at the bottom of groove decreased with the increase of laser micro-engraving times. It was difficult to observe the sheet structure. Furthermore, the convex structures became smaller and more individual than before. The number of the particles covered on the convex structure increased significantly. As the laser micro-engraving times further increased, the strip grooves remained unchanged (Figure 7b,d). But the sheet microstructure at the bottom of groove disappeared. Additionally, humps were formed on the both sides of the strip groove. Due to the increase of laser micro-engraving times, the number of particles covered on the microstructure surface further increased.

**Figure 8:**
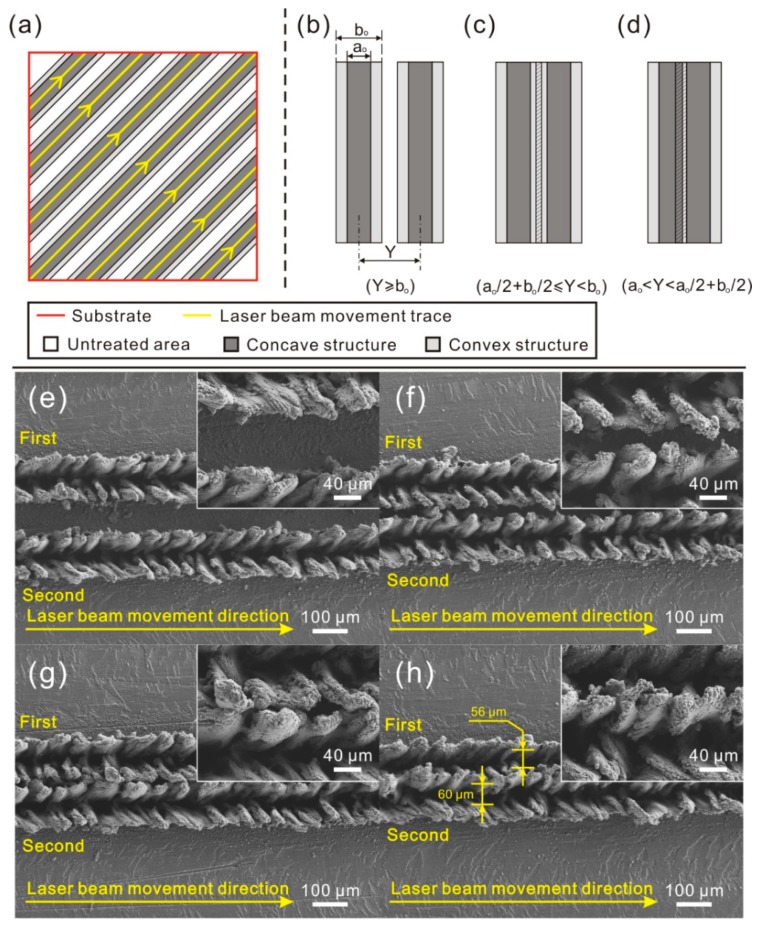
(**a**) Schematic of laser beam movement trace during laser micro-engraving; (**b**–**d**) relationship between microstructure and texture spacing; (**e**–**h**) SEM image of the strip microstructure with different texture spacing (laser beam pulse frequency of 6000 Hz, laser beam scanning speed of 0.15 m/s).

According to the treatment process, the laser beam travels along a designated track. From Figure 8a, it is found that the microstructure on the surface is composed of numerous parallel strips. The spacing of strip microstructure (texture spacing) may greatly affect the morphology of the as-prepared microstructure. In order to reveal the effect of texture spacing on the roughness structure, the morphology of the strip microstructures with different texture spacing was observed and analyzed. As shown in Figure 8b–d, some conclusions could be drawn on the strip microstructures with different texture spacing: (1) Strip microstructures do not contact with each other (Figure 8b); (2) Convex structures of strip microstructures contact with each other (Figure 8c); (3) Convex structures contact with the concave structure of other strip microstructures (Figure 8d). As shown in Figure 8e,f, since there was no contact between the microstructures, the strip microstructure on the surface had no change with the decrease of texture spacing. Reduction was only observed in the width of the untreated surface between two strip microstructures. When the convex structures of strip microstructures were contacting with each other (Figure 8g), the strip grooves were not affected. However, the convex structures of strip microstructures were flocked together, resulting in the formation of peak-like microstructure. As the texture spacing further decreased (Figure 8h), no obvious change was found in the two strip grooves, but the width of the first-fabricated strip grooves (56 μm) was a little shorter than that of the second one (60 μm). From the SEM image presented in the inset of Figure 8h, interconnected sheet microstructures appeared, instead of the strip peak-like structure. This is because that some part of the convex structure of the first strip microstructure is melted again when second strip microstructure is prepared. Meanwhile, this melted material gathers with the melted material squeezed from the second strip groove in the overlap zone, resulting in the decrease in the width of the first strip groove.

### 3.3. Morphology Regulation Rule

**Figure 9:**
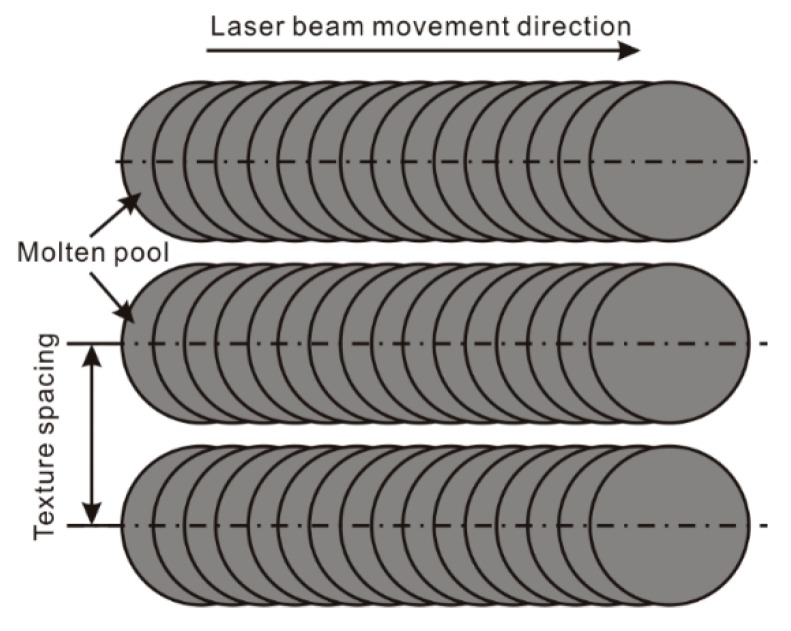
Schematic of overlap degree of melting zone.

According to Figure 9, due to the effect of the laser beam pulse frequency on the number of laser beam irradiations per second and the energy of each laser beam pulse, the diameter and distance of the molten pool are related to the laser beam pulse frequency. Additionally, the laser beam scanning speed can affect the distance between each molten pool directly. Therefore, it can be speculated that the laser beam pulse frequency and scanning speed of laser micro-engraving treatment have a great influence on the morphology of the as-prepared microstructure. In order to get an insight into the effect of laser parameters on the microstructure, systematic investigation was performed on the morphology of the strip microstructures treated by different laser beam scanning speed and pulse frequency.

**Figure 10:**
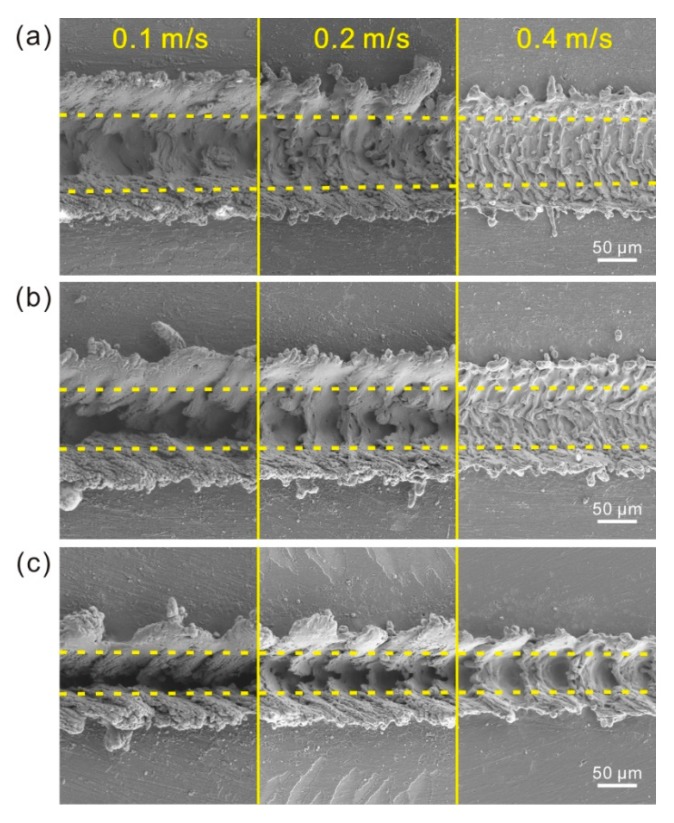
SEM image of the strip microstructure prepared by different laser beam scanning speed: (**a**) 3000 Hz; (**b**) 5000 Hz; (**c**) 8000 Hz.

As shown in Figure 10a, under the laser beam pulse frequency of 3000 Hz, as the laser beam scanning speed increased, the concave structure maintained its strip groove. However, the depth of the groove significantly decreased. Simultaneously, the size of the convex structure on the both sides of the strip groove reduced. The convex structure became more individual and irregular, leading to the transformation of convex microstructure into individual hump. The amount of fold microstructure at the bottom of the groove increased with the increase of scanning speed as well. Furthermore, the number of the particles on the untreated surface became fewer. When the laser beam pulse frequency was 5000 Hz and 8000 Hz, similar phenomenon was observed (Figure 10b,c). The results indicate that laser beam scanning speed has a great influence on the morphology and size of as-prepared microstructure (convex and concave structures). Because of the unchanged energy of laser beam, the diameter of hemispherical molten pool almost maintains a constant value as laser beam scanning speed increases. But the overlap degree of molten pool decreases. Thus, the melt degree of the material in the depth direction (−Z-axis) reduces dramatically. Moreover, the effect of laser beam impact on melted material per unit area and the degree of blasting of melted material become weak as scanning speed increases. As shown in Figure 10c, due to the decrease of overlap degree of molten pool, the concave structure transformed from strip groove to successive pits as the scanning speed increased to 0.4 m/s, which was consistent with the aforementioned results.

**Figure 11:**
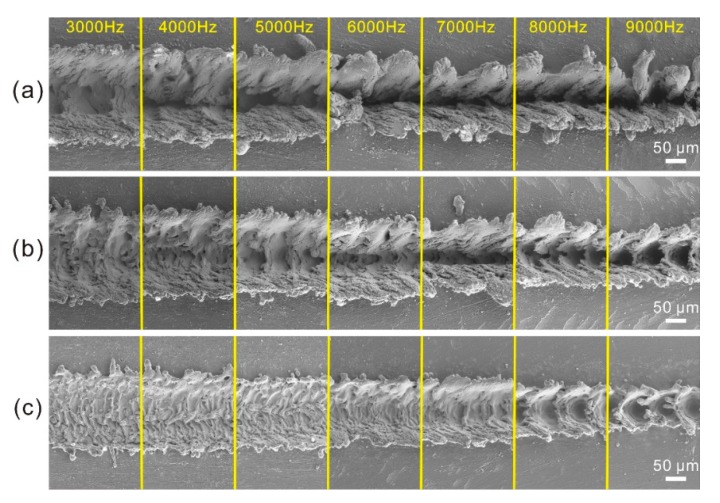
SEM image of the strip microstructure on Al alloy surface prepared by different laser beam pulse frequency: (**a**) 0.1 m/s; (**b**) 0.20 m/s; (**c**) 0.4 m/s.

The morphologies of the microstructures treated with different laser beam pulse frequencies are shown in Figure 11. When the laser beam scanning speed was 0.1 m/s, strip groove was observed on Al alloy surface (Figure 11a). Nevertheless, the width of the strip groove and the convex structure on both sides of the strip groove decreased greatly with the increase of laser beam pulse frequency. In contrast, the depth of the strip groove increased. Meanwhile, the number of fold microstructures at the bottom of the strip groove and the irregular protrusions on the microstructure reduced dramatically with the increase of laser beam pulse frequency, meaning that the convex and concave microstructures of the surface become more and more regular. When the laser beam scanning speeds were 0.20 m/s and 0.4 m/s respectively, the morphology of the microstructure exhibited similar change as the laser beam pulse frequency increased. The above results indicate that the laser beam pulse frequency can affect the microstructures of the surface greatly. According to the mechanism of laser micro-engraving treatment, the energy of each laser beam reduces with the increase of laser beam pulse frequency. Thus, the diameter of molten pool has a decrease, leading to the decrease of width of the strip groove. Meanwhile, the distance between each molten pool decreased with the increase of laser beam pulse frequency. The decrease of the diameter of molten pool and the distance between each molten pool mutually resulted in the raise of melt degree of material in depth direction (−Z-axis). Furthermore, the reduction of distance between each molten pool enhanced the effect of laser beam impact on the melted material per unit area. Squeezing action was improved. Hence, the depth of the groove increased dramatically. The microstructure of the surface became more and more regular. Actually, if the decrease rate of the distance of each molten pool is much higher than that of the diameter of molten pool, the overlap degree of molten pool will increase with the increase of laser beam pulse frequency. In contrast, the overlap degree of molten pool will decrease by increasing laser beam pulse frequency. Thus, the overlap degree of the molten pool increased and then decreased with the increase of laser beam pulse frequency from 3000 to 9000 Hz. As shown in Figure 11c, when the laser beam pulse frequency increased to 8000 Hz, due to the one-time overlap of each molten pool with others, the concave structure became successive pits.

To further understand the effect of laser parameters on the as-prepared microstructure and to achieve the controllable preparation of microstructure on Al alloy surface, the following part explored the variations of the geometrical dimensions of strip microstructure with laser beam pulse frequency and scanning speed. The dimension of the microstructures of the treated surface shown in Figure 11 was measured on a Contour Elite I 3D optical surface profiler. Measurements were conducted five times and the mean value was adopted.

**Figure 12:**
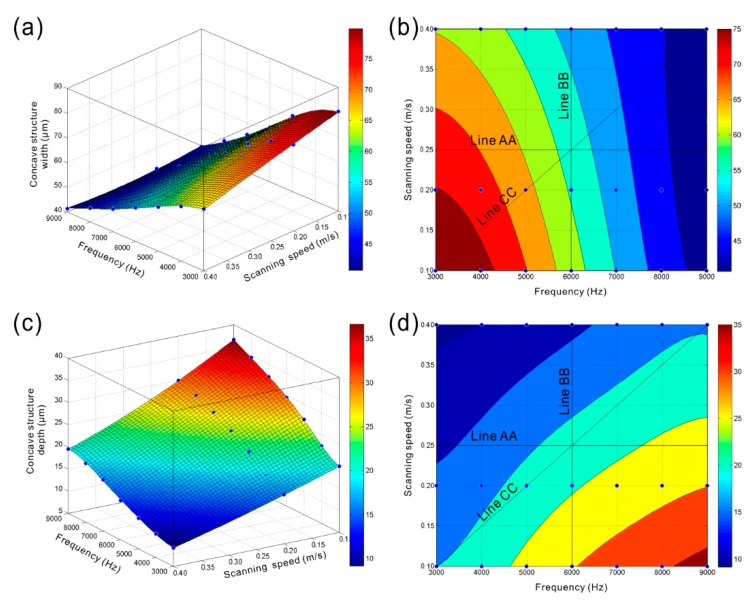
(**a**,**b**) Variations of concave structure width of the strip microstructure with laser beam pulse frequency and scanning speed; (**c**,**d**) Variations of depth of concave structure of the strip microstructure with laser beam pulse frequency and scanning speed.

Figure 12a,b present the variations of width of concave structure (pit or groove) of the strip microstructure with laser beam pulse frequency and scanning speed. Obviously, as the laser beam pulse frequency increased, the width of concave microstructure reduced significantly (line AA shown in Figure 12b). However, the width of concave structure had a slight decrease with the increase of laser beam scanning speed (line BB shown in Figure 12b). The aforementioned results indicate that the overlap degree of molten pool has little effect on the width of concave microstructure. The width is mainly affected by the diameter of molten pool. The width of concave microstructure of the strip structure would dramatically decrease as the laser beam pulse frequency and scanning speed increased simultaneously (line CC shown in Figure 12b). From Figure 12c,d, opposite variation could be found. The depth of concave structure gradually increased with the increase of laser beam pulse frequency (line AA shown in Figure 12d). In contrast, the depth of concave structure of the strip microstructure declined as the laser beam scanning speed rose (line BB shown in Figure 12d). According to the above research, both the increase of overlap degree and the size of hemisphere molten pool can increase the depth of concave structure of the strip microstructure. Therefore, it can be concluded that the overlap degree of molten pool has more profound effect on the depth of the concave structure than the size of hemispherical molten pool. From the line CC shown in Figure 12d, firstly, the depth of concave microstructure had a slight increase with the increase of laser beam pulse frequency and scanning speed. Then, due to the slight decrease of overlap degree of molten pool, the depth reduced as the laser beam pulse frequency increased to 8000 Hz.

**Figure 13:**
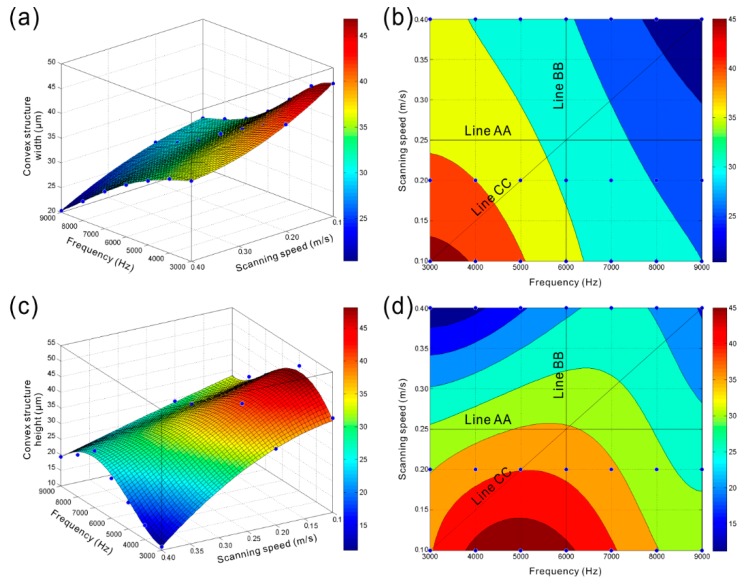
(**a**,**b**) Variations of convex structure width of the strip microstructure with laser beam pulse frequency and scanning speed; (**c**,**d**) Variations of convex structure height of the strip microstructure with laser beam pulse frequency and scanning speed.

The variations of convex structure width of the strip microstructure with laser beam pulse frequency and scanning speed are shown in Figure 13a,b. Obviously, the width of convex structure decreased with the increase of laser beam pulse frequency (line AA shown in Figure 13b). It is considered that, due to the increase of melt degree of material in depth direction (−Z-axis) and the improvement of squeezing action, more melted material is squeezed out from the molten pool. As the depth of the concave structure increases gradually, the accumulation degree of melted material in the opposite direction of depth (+Z-axis) increases. Instead, the accumulation degree of melted material in the direction vertical to the laser beam movement direction has a decrease, leading to the decrease of convex structure width of the strip microstructure. As shown in Figure 13b, the convex structure width decreased slowly with the increase of laser beam scanning speed, which resulted from the similar size of each molten pool. Actually, the decrease of overlap degree of the molten pool will lead to the reduction in the quantity of melted material per unit area. Meanwhile, the effect of the laser beam impact on the melted material per unit area becomes weak. From the line CC shown in Figure 13b, as the laser beam pulse frequency and scanning speed increased at the same time, the width of convex structure would decrease sharply. The width decreased from 46.830 μm (laser beam pulse frequency of 3000 Hz, laser beam scanning speed of 0.1 m/s) to 20.320 μm (laser beam pulse frequency of 9000 Hz, laser beam scanning speed of 0.4 m/s). Figure 13c,d show the variations of convex structure height of the strip microstructure with laser beam pulse frequency and scanning speed. Clearly, when the laser beam scanning was 0.25 m/s, the height of convex structure increased with the increase of laser beam pulse frequency from 3000 Hz to 6000 Hz. As laser beam pulse frequency further increased, the height of the convex structure decreased. A similar phenomenon could be observed under different scanning speeds. According to the above-obtained results, the increase of the height of convex structure resulted from the increase of melted material quantity per unit area and the accumulation degree of melted material in the opposite direction of depth (+Z-axis). When the laser beam pulse frequency further increases, though the melt degree of material in depth direction further increases, the size of hemispherical molten pool reduces dramatically, which further decreases the quantity of melted material per unit area. Moreover, due to the further increase of concave structure depth of the strip microstructure, the accumulation of melted material in the opposite direction of depth (+Z-axis) becomes more difficult. Thus, the height of the convex microstructure decreases. According to the line BB shown in Figure 13d, because of the reduction of melted material per unit area and the weaker effect of the laser beam impact on the melted material, the height of convex structure showed a significant decrease with the increase of laser beam scanning speed. From the line CC shown in Figure 13d, it could be speculated that the height of the convex structure increased and then gradually decreased with the increase of laser beam pulse frequency and scanning speed.

### 3.4. Roughness Structure Predictive Model

On the basis of the aforementioned results and by the method of data fitting, roughness structure predictive model was established, which clarified the relationship between the geometrical dimensions of the microstructure on Al alloy surface and laser parameters of laser micro-engraving treatment. The equations are shown as follows:

The width of concave structure of the constructed microstructure-D_1_:

(1) $$\begin{array}{ll} D_{1}= & 42.37+0.03465x-11.49y-9.067\times 10^{-6}x^{2}-0.02494xy+20.75y^{2}+7.972\\ & \times 10^{-10}x^{3}+6.06\times 10^{-6}x^{2}y-2.076\times 10^{-2}xy^{2}-2.366\times 10^{-14}x^{4}-3.97\\ & \times 10^{-10}x^{3}y+2.802\times 10^{-6}x^{2}y^{2}, \end{array}$$

The depth of concave structure of the constructed microstructure-L_1_:

(2) $$\begin{array}{ll} L_{1}= & 85.46-0.05739x-196.5y+1.93\times 10^{-5}x^{2}+0.1115xy+181.2y^{2}-2.884\times 10^{-9}x^{3}\\ & -2.824\times 10^{-5}x^{2}y-8.749\times 10^{-2}xy^{2}+2.047\times 10^{-13}x^{4}+2.995\\ & \times 10^{-9}x^{3}y+1.244\times 10^{-54}x^{2}y^{2}-5.556\times 10^{-18}x^{5}-1.197\\ & \times 10^{-13}x^{4}y-4.815\times 10^{-10}x^{3}y^{2}, \end{array}$$

The width of convex structure of the constructed microstructure-D_2_:

(3) $$\begin{array}{ll} D_{2}= & 14.96+0.03597x+5.632y-1.059\times 10^{-5}x^{2}-0.09931xy+185.3y^{2}+1.096\\ & \times 10^{-9}x^{3}+3.313\times 10^{-5}x^{2}y-2.55\times 10^{-2}xy^{2}-3.496\times 10^{-14}x^{4}-3.718\\ & \times 10^{-9}x^{3}y-2.151\times 10^{-6}x^{2}y^{2}-3.333\times 10^{-19}x^{5}+1.393\\ & \times 10^{-13}x^{4}y+2.13\times 10^{-10}x^{3}y^{2}, \end{array}$$

The height of convex structure of the constructed microstructure-H_2_:

(4) $$\begin{array}{ll} H_{2}= & -40.55+0.03453x+531.7y-1.173\times 10^{-6}x^{2}-0.2764xy-642.9y^{2}-6.223\\ & \times 10^{-10}x^{3}+4.138\times 10^{-5}x^{2}y+0.1707xy^{2}+4.814\times 10^{-14}x^{4}-1.97\\ & \times 10^{-9}x^{3}y-1.115\times 10^{-5}x^{2}y^{2}, \end{array}$$

where *x* and *y* represent laser beam pulse frequency (Hz) and laser beam scanning speed (m/s), respectively.

**Figure 14:**
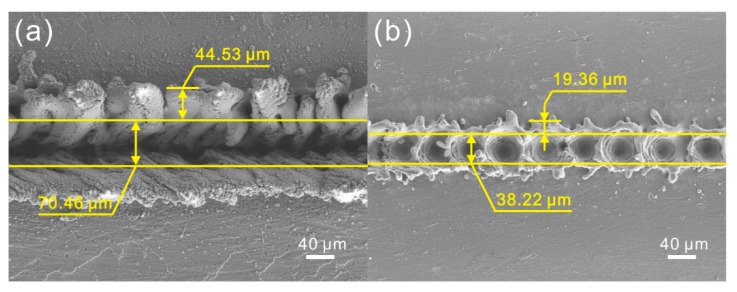
SEM images of the microstructure on Al alloy surface treated by laser micro-engraving: (**a**) laser beam pulse frequency of 5000 Hz, laser beam scanning speed of 0.05 m/s; (**b**) laser beam pulse frequency of 10,000 Hz, laser beam scanning speed of 0.4 m/s.

According to the mathematical relationship, the width and depth of concave structure of the microstructure on Al alloy surface treated under laser beam pulse frequency of 5000 Hz and scanning speed of 0.05 m/s were calculated to be 70.06 μm and 28.80 μm, respectively (Table 1). And the width and height of the convex structure were 42.23 μm and 51.80 μm. As shown in Figure 14a, the widths of concave and convex microstructures prepared under aforementioned laser parameters were 70.46 μm and 44.53 μm, respectively. Meanwhile, the depth of the concave structure (30.04 μm) and the height of convex structure (50.10 μm) measured by the optical surface profilers are exhibited in Table 1. It could be found that the calculated dimensions of the microstructure were highly consistent with the results of actual measurement. For the microstructure treated under a laser beam pulse frequency of 10,000 Hz and a scanning speed of 0.4 m/s, similar phenomenon could be observed (Table 1 and Figure 14b). The results indicate that the prepared microstructures can be mathematically calculated by the established model instead of actual measurement. Moreover, the geometrical dimensions of the microstructure on Al alloy surface treated by laser micro-engraving can be predicted and regulated by the model in the future, which promotes achieving the controllable preparation of microstructure on Al alloy.

### 3.5. Chemical Composition

**Figure 15:**
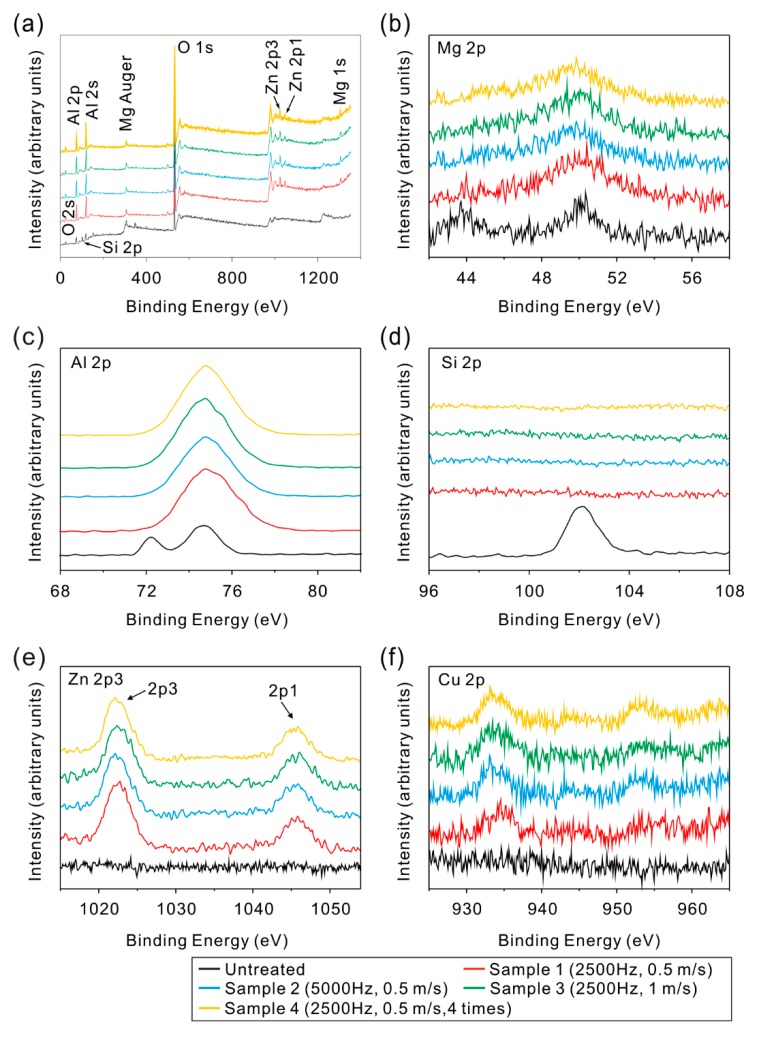
XPS spectra of the microstructure surface prepared by laser micro-engraving: (**a**) survey; (**b**) Mg 2p; (**c**) Al 2p; (**d**) Si 2p; (**e**) Zn 2p3; (**f**) Cu 2p.

In addition to the change of microstructure morphology, the composition may change. In order to understand the change trend of composition, X-ray photoelectron spectroscopy (XPS) was carried out to investigate the composition of the as-prepared microstructure surfaces. From the XPS spectra shown in Figure 15, compared with untreated surface, zinc and copper was detected on the treated microstructure surfaces. Meanwhile, silicon disappeared. As shown in Figure 15c, both the peaks at 72.4 eV (Al_2_O_3_) and 74.7 eV (pure aluminum) were detected on the untreated surface. However, the peak at 72.4 eV disappeared after laser micro-engraving treatment, which indicated that the pure aluminum was completely oxidized to Al_2_O_3_ on the surface. From the element contents of the microstructure surfaces shown in Table 2, the aluminum content decreased dramatically after laser treatment. In contrast, the content of oxygen significantly increased. The results in this research have a good agreement with previous studies [32]. However, the chemical composition of microstructure surfaces treated with different process parameters almost remained the same, indicating that the process parameters (frequency, scanning speed and scanning times) have little effect on the composition of the as-prepared microstructure. The difference of the element content results from the change of surface morphology. Nevertheless, since the detection depth of XPS is less than 20 nm, the effect of surface morphology difference on the content of the elements measured by XPS is slight, which shows that the change is not readily obvious.

## 4. Conclusions

In order to realize controllable preparation of microstructure on Al alloy, a simple, convenient, high-efficient, low-cost and suitable for engineering application micro-scale roughness structure construction approach (laser micro-engraving) was developed. Systematic investigation was performed on the as-prepared microstructure on Al alloy surface formed by laser micro-engraving. The morphology and formation mechanism of the microstructure under different process conditions were studied. Meanwhile, the effect of laser parameters on morphology and geometrical dimensions of microstructure was investigated. The effect rule and mechanism were revealed. Additionally, a mathematical relationship model between the geometrical dimensions of as-prepared microstructure and laser parameters of laser micro-engraving treatment was established. Furthermore, the influence of laser parameters on chemical composition of the microstructure was clarified. Controllable preparation of microstructure is realized, showing that expected microstructure can be prepared rapidly and accurately instead of suffering from longtime experimental investigation in the future. The main findings are as follows:

(1) During laser micro-engraving, material of surface layer is melted and vaporized. Meanwhile, a part of the melted material in molten pool is squeezed and piled up by laser beam impact, which leads to the formation of concave microstructure and convex microstructure. Furthermore, due to the high pressure difference between vaporized material and atmospheric, melted material will blast and splash, resulting in the production of irregular particles that is randomly distributed on the microstructure.

(2) Due to the significant effect of overlap degree of molten pool on the melt degree of material, laser beam impact degree and blasting degree of melted material, the morphology of the as-prepared microstructure (especially concave microstructure) is affected greatly. When each molten pool does not overlap with others, successive individual pits are constructed. When each molten pool overlaps with others for one-time, successive overlapping pits form. As the overlap degree of molten pool further increases (overlapping with others for more than one time), the successive pits become grooved.

(3) The processing times have little influence on the morphology of the constructed microstructure. However, the morphology is affected by the spacing of the microstructures significantly. The morphology change degree depends on the contact degree of the adjacent microstructures.

(4) Under different process conditions of laser micro-engraving treatment, not only can the microstructure be prepared on Al alloy surface, but also nanostructure can be formed.

(5) Because of the influence of laser beam pulse frequency and scanning speed on the diameter and distance of the molten pools, the morphology and geometrical dimensions of as-prepared microstructure are greatly affected by laser parameters.

(6) The laser parameters have little effect on the chemical composition of microstructure. The chemical composition of the microstructure fabricated by different laser parameters almost remains the same.

(7) Roughness structure predictive model is successfully established, which can be used to predict and regulate the geometrical dimensions of the microstructure on Al alloy surface treated by laser micro-engraving in the future.

## Figures and Tables

**Table 1:** Dimensions of the microstructure.

		Width of Concave Structure	Depth of Concave Structure	Width of Convex Structure	Height of Convex Structure
Laser beam pulse frequency of 5000 Hz, laser beam scanning speed of 0.05 m/s	Model calculation	70.06 μm	28.80 μm	42.23 μm	51.80 μm
	Actual measurement	70.46 μm	30.04 μm	44.53μm	50.10 μm
Laser beam pulse frequency of 10,000 Hz, laser beam scanning speed of 0.4 m/s	Model calculation	36.95 μm	16.97 μm	17.45 μm	12.68 μm
	Actual measurement	38.22 μm	18.11 μm	19.36 μm	10.37 μm

**Table 2:** Element contents of the microstructure surface calculated from XPS survey spectra.

	Al (At. %)	O (At. %)	Mg (At. %)	Si (At. %)	Zn (At. %)	Cu (At. %)
Untreated surface	66.80	29.64	0.89	2.67	0	0
Sample 1	31.76	64.34	2.30	0	1.28	0.32
Sample 2	31.17	64.91	2.15	0	1.37	0.40
Sample 3	32.39	63.57	2.35	0	1.31	0.38
Sample 4	31.49	64.77	1.98	0	1.40	0.36
